# Cardiovascular Outcomes of Sitagliptin in Type 2 Diabetic Patients with Acute Myocardial Infarction, a Population-Based Cohort Study in Taiwan

**DOI:** 10.1371/journal.pone.0131122

**Published:** 2015-06-26

**Authors:** Szu-Heng Wang, Dong-Yi Chen, Yu-Sheng Lin, Chun-Tai Mao, Ming-Lung Tsai, Ming-Jer Hsieh, Chung-Chuan Chou, Ming-Shien Wen, Chun-Chieh Wang, I-Chang Hsieh, Kuo-Chun Hung, Tien-Hsing Chen

**Affiliations:** 1 Department of Medical education, Chang Gung Memorial Hospital, Taoyuan, Taiwan; 2 College of Medicine, Chang Gung University, Taoyuan, Taiwan; 3 Division of Cardiology, Department of Internal Medicine, Chang Gung Memorial Hospital, Taoyuan, Taiwan; 4 Division of Cardiology, Department of Internal Medicine, Chang Gung Memorial Hospital, Chiayi, Taiwan; 5 Heart Failure Center, Division of Cardiology, Department of Internal Medicine, Chang Gung Memorial Hospital, Keelung, Taiwan; 6 Department of cardiology, Chang Gung Memorial Hospital, Xiamen, China; Osaka University Graduate School of Medicine, JAPAN

## Abstract

**Background:**

The cardiovascular safety and efficacy of sitagliptin, a dipeptidyl peptidase 4 (DPP-4) inhibitor, in type 2 diabetic patients after acute myocardial infarction (AMI) has so far remained uncertain.

**Methods:**

We analyzed data from the National Health Insurance Research Database (NHIRD), a government-operated, population-based database, from March 1st, 2009 to December 31st, 2011. Type 2 diabetic patients hospitalized for AMI were included in our study. We compared subjects using sitagliptin with comparison group to evaluate its cardiovascular safety and efficacy. The primary endpoint was a composite of cardiovascular death, myocardial infarction, and ischemic stroke.

**Results:**

We identified a total of 3,282 type 2 diabetic patients hospitalized for AMI (mean follow-up 1.15 years). Of these patients, 547 (16.7%) who were exposed to sitagliptin were defined as the sitagliptin group and 2,735 (83.3 %) who did not use sitagliptin were the comparison group. The incidence of primary composite cardiovascular outcomes was 9.50 per 100 person-years in the sitagliptin group and was 9.70 per 100 person-years in the comparison group (hazard ratio (HR), 0.97; 95% CI, 0.73–1.29, P=0.849). Compared to the non-sitagliptin group, the sitagliptin group had similar risks of all-cause mortality, hospitalization for heart failure (HF) or percutaneous coronary intervention (PCI) with a HR of 0.82 (95% CI, 0.61–1.11, P=0.195), 0.93 (95% CI, 0.67–1.29, P=0.660), and 0.93 (95% CI, 0.75–1.14, P=0.473), respectively.

**Conclusion:**

The use of sitagliptin in type 2 diabetic patients with recent AMI was not associated with increased risk of adverse cardiovascular events.

## Introduction

Type 2 diabetes mellitus (T2DM) is associated with elevated risk of cardiovascular disease; more than half of patients with diabetes die of cardiovascular complications [1,2]. Diabetic patients who have yet to develop myocardial infarction have comparable cardiovascular risk to that of non-diabetic patients with a prior myocardial infarction. Diabetic patients who have a history of myocardial infarction are at an even higher risk, with a seven-year AMI incidence of 45% [3]. Improved glycemic control has been shown to reduce the risk of microvascular complications of T2DM, but studies have failed to demonstrate that glycemic control reduces the risk of macrovascular events [4–6]. Concerns about adverse cardiovascular events with antidiabetic agents indicate a clinical need to identify the cardiovascular safety and benefit of antihyperglycemic agents [7,8].

Sitagliptin is an orally administered dipeptidyl peptidase-4 (DPP-4) inhibitor that exerts antihyperglycemic effects by increasing the availability of incretin hormones, which in turn modulates pancreatic islet hormone secretion [9,10]. Some studies have revealed a decreased risk of adverse cardiovascular events in DPP-4-treated subjects [11] whereas others suggest a neutral effect on cardiovascular events [12–14]. Moreover, results from observational studies have shown that sitagliptin may increase cardiovascular risk [15], especially in patients with chronic kidney disease [16]. As a result, there remains much speculation about the cardiovascular benefit and potential risks of this medication.

This nationwide, prospective cohort study aimed to examine sitagliptin use and cardiovascular outcomes in patients with T2DM after AMI. Secondary safety outcomes were also considered.

## Methods

### Data Source

We conducted this nationwide population-based cohort study using Taiwan’s National Health Insurance Research Database (NHIRD), a government-operated, population-based database derived from the claims data of Taiwan’s National Health Insurance program, covering 99.19% of the population [17]. The NHIRD database provides comprehensive and accurate records of beneficiaries, including ambulatory visits, inpatient care, disease diagnosis codes, and medication prescriptions. The accuracy and validity of NHIRD data has been previously confirmed [18–20]. The Ethics Institutional Review Board of Chang Gung Memorial Hospital approved the study.

### Study Population

Patients with a diagnosis of type 2 diabetes (*International Classification of Diseases*, *Ninth Revision*, *Clinical Modification* [ICD-9-CM] code 250.xx) were included in this study. We identified patients who were hospitalized for AMI (ICD-9-CM code 410.xx) between March 1st, 2009 and December 31st, 2011. The index hospitalization was defined as the date on which patient was admitted for AMI. Patients’ baseline characteristics, such as gender and age, were considered. We also identified baseline comorbidities, medication prescription, and previous medical procedures, such as percutaneous coronary intervention (PCI) and coronary artery bypass grafting (CABG).

**Fig 1:**
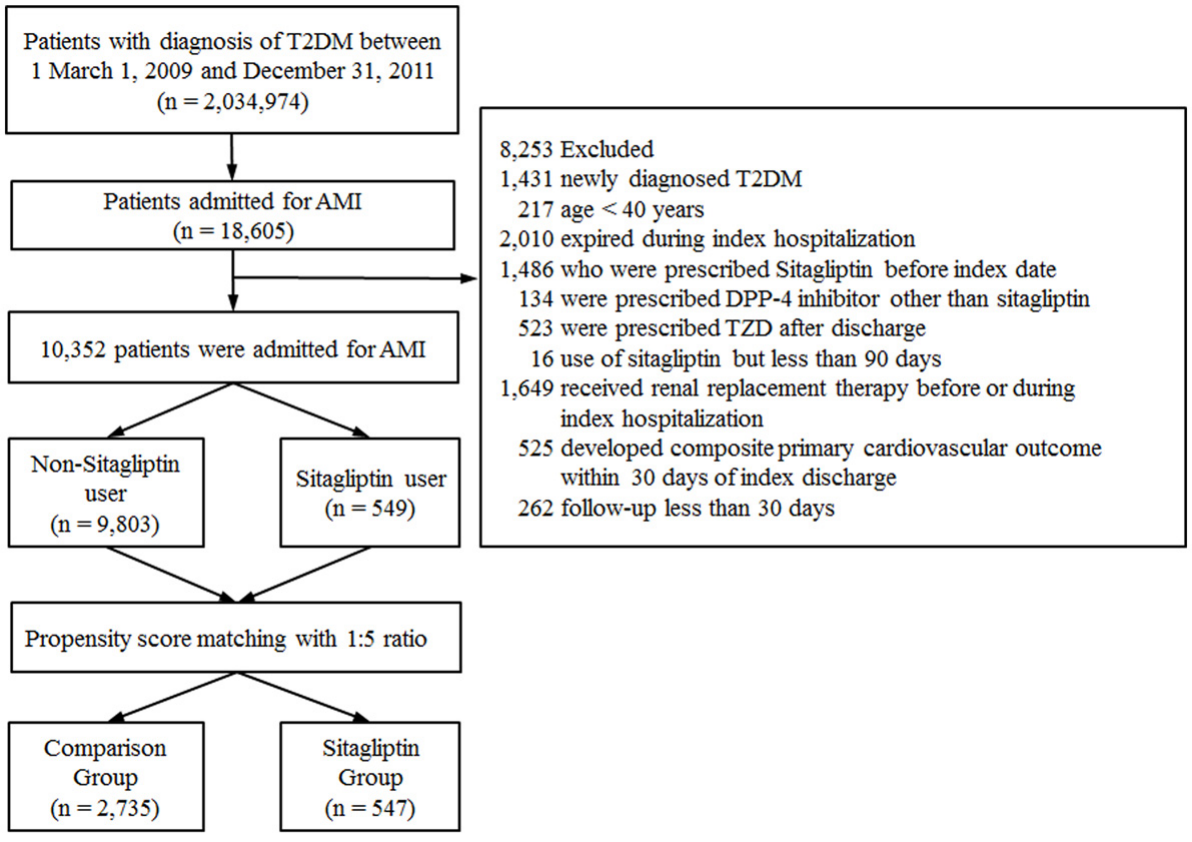
Flow chart of patient enrollment. T2DM patients hospitalized with a diagnosis of AMI were included in our analysis after relevant exclusions (T2DM = type 2 diabetes mellitus, AMI = acute myocardial infarction, DPP-4 = dipeptidyl peptidase 4, TZD = thiazolidinediones).

Patients were excluded if they met any of the following criteria. (Fig 1): (1) age < 40 years; (2) expired during index hospitalization for AMI; (3) received sitagliptin treatment before index hospitalization; (4) use of thiazolidinediones or other DPP-4 inhibitors; (5) received renal replacement therapies; (6) developed a composite primary cardiovascular endpoint (defined as death, AMI or ischemic stroke) within 30 days of discharge; (7) were followed for less than 30 days after the index hospitalization; and (8) was diagnosed with T2DM during index hospitalization (defined as patient who did not use antihyperglycemic agents prior to index hospitalization).

### Exposure to Sitagliptin and Concomitant Medications

Sitagliptin exposure was based on computer-based prescription claims after the index hospitalization. We defined patients who received a prescription of sitagliptin for 90 consecutive days following index discharge as the sitagliptin group, while those who did not receive sitagliptin were considered the comparison cohort. Sitagliptin dosages were prescribed according to Taiwan’s National Health Insurance regulations, which were 100mg, 50mg and 25mg daily for patients with an estimated glomerular filtration rate (eGFR) of over 50 ml/min, between 30 to 50 ml/min and below 30 ml/min, respectively. We used NHIRD claims data from ambulatory visits to obtain data on concomitant medication use in each patient. The following pharmaceutical agents were considered: metformin, sulfonylurea, insulin, angiotensin-converting-enzyme inhibitors, angiotensin II receptor blockers, aspirin, antiplatelets, calcium channel blockers, diuretics and statins.

### Study Endpoints and Covariates

Baseline comorbidities were identified by ICD-9-CM diagnosis codes and medications during index hospitalization (see S1 Table). We defined primary outcomes as composite events of cardiovascular death, myocardial infarction or ischemic stroke. The definition of cardiovascular death met the criteria of the Standardized Definitions for End Point Events in Cardiovascular Trials draft by the United States Food and Drug Administration [21]. Death and causes of death were collected from registry data of the NHIRD [22]. Other secondary outcomes of interest were death by any cause, hospitalization for heart failure, coronary revascularization, pancreatitis, hypoglycemia and diabetic ketoacidosis (DKA) or hyperosmolar hyperglycemic state (HHS).

### Statistical Analysis

Propensity score matching (PSM) was used to reduce potential confounding and selection biases because patients in this study were not randomly assigned to treatment with sitagliptin [23]. The sitagliptin cohort was matched with the comparison cohort according to a 1:5 ratio in terms of patient’s characteristics, baseline comorbidities, medication prescribed 90 days since index hospitalization (listed in Tables 1 and 2), and index year and month using the PSM method. The PSM matching algorithm was based on the nearest-neighbor method in which the treated and control subjects were randomly ordered according to the estimated propensity score, then the first treated subject was selected and the corresponding control subject(s) were found and matched with the closest propensity score. In addition, the PSM matching was performed with the caliper radius, which the control subjects within a predefined amount of the estimated propensity score (set as 0.5 sigma) are selected and matched [24]. The matching procedure was performed with SAS Version 9.3 (SAS Institute, Cary, NC).

**Table 1:** Baseline clinical characteristics of the study patients.

Characteristics	Sitagliptin (n = 547)	Comparison (n = 2,735)	*P*
Age, year	66.0±12.2	65.9±12.1	0.911
Age≧75 years	149 (27.2)	725 (26.5)	0.724
Gender			0.909
Male	350 (64.0)	1,743 (63.7)	
Female	197 (36.0)	992 (36.3)	
Previous myocardial infarction	20 (3.7)	108 (3.9)	0.747
Previous cerebral vascular accident	73 (13.3)	341 (12.5)	0.573
Comorbidity			
Coronary artery disease	413 (75.5)	2,069 (75.6)	0.942
Chronic kidney disease	48 (8.8)	232 (8.5)	0.823
Peripheral arterial disease	27 (4.9)	137 (5.0)	0.943
Hypertension	411 (75.1)	2,076 (75.9)	0.702
Heart failure	181 (33.1)	920 (33.6)	0.804
Dyslipidemia	335 (61.2)	1,703 (62.3)	0.652
Previous PCI	367 (67.1)	1,844 (67.4)	0.881
Previous CABG	4 (0.7)	18 (0.7)	0.848
Follow-up days	422.2±263.0	418.7±274.1	0.783
Propensity score, %	7.3±3.7	7.3±3.6	0.784

Values are mean ± SD or n (%); PCI = percutaneous coronary intervention; CABG = coronary artery bypass grafting.

**Table 2:** Concomitant medications use within 90 days of index discharge.

Medications	Sitagliptin (n = 547)	Comparison (n = 2,735)	*P*
ACEI or ARB	380 (69.5)	1,947 (71.2)	0.419
Aspirin	453 (82.8)	2,281 (83.4)	0.738
Antiplatelet agents	492 (89.9)	2,448 (89.5)	0.759
Beta-blockers	389 (71.1)	1,975 (72.2)	0.602
Calcium-channel blockers	154 (28.2)	791 (28.9)	0.717
Diuretics	234 (42.8)	1,201 (43.9)	0.626
Statins	361 (66.0)	1,835 (67.1)	0.619
Insulin	86 (15.7)	442 (16.2)	0.799
Metformin	269 (49.2)	1,341 (49.0)	0.950
Sulfonylurea	322 (58.9)	1,595 (58.3)	0.812

Values are n (%); ACEI = angiotensin-converting-enzyme inhibitor; ARB = angiotensin II receptor blocker.

We compared clinical characteristics between study groups (sitagliptin and comparison groups) by chi-square test for categorical variables and by independent sample t-test for continuous variables. We used Cox proportional hazards models to compare time to first occurrence of a predefined primary or secondary outcome following index hospitalization between study groups, adjusting the propensity score. We estimated the survival rates of a predefined period (i.e. three months and one year) for each study group, depicted with the Kaplan-Meier method. On the other hand, we reported the incidence density (per 100 person-years) as to the event at the complete course. All data analysis was conducted using IBM SPSS software version 22 (IBM SPSS Inc, Chicago, Illinois).

## Results

### Study Population

A total of 3,282 patients with type 2 diabetes who were hospitalized for AMI between March 2009 and December 2011 were identified for the study cohort. Of these patients, 547 (16.7%) were in the Sitagliptin group and 2,735 (83.3%) matched subjects were in the comparison group. The mean age for the overall cohort was 65.9 years (SD = 12.1 years). The mean follow-up period was 1.15 years (SD = 0.75 years), and the maximum follow-up time was 2.84 years. The two study groups were well matched with respect to baseline characteristics, comorbidities, follow-up period and concomitant medications.

### Cardiovascular Outcomes

**Fig 2:**
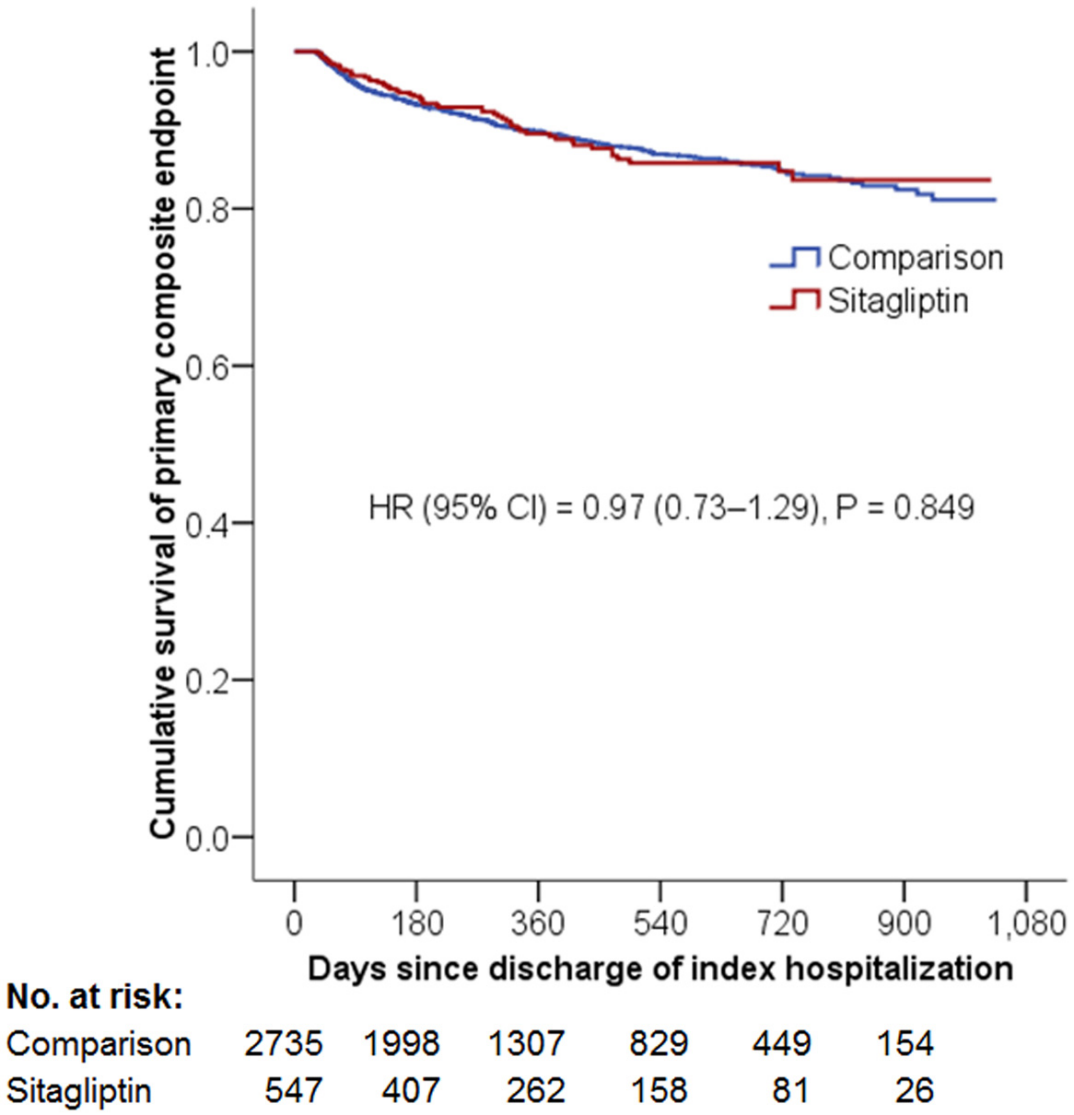
Cumulative Kaplan-Meier survival estimates of the time to primary composite endpoint. The primary endpoint was a composite of myocardial infarction, ischemic stroke, and cardiovascular deatFTh. No significant differences in the primary composite outcomes were observed between the two study groups after a mean follow-up of 14 months.

The incidence of composite primary cardiovascular outcome was 9.50 per 100 person-years in the Sitagliptin group and 9.70 per 100 person-years in the comparison group (HR = 0.97; 95% CI, 0.73–1.29, P = 0.849) (Fig 2). The risk of recurrent myocardial infarction (HR = 1.07; 95% CI, 0.72–1.59, P = 0.738), ischemic stroke (HR = 1.30; 95% CI, 0.75–2.26, P = 0.346) or cardiovascular death (HR = 0.65, CI, 0.39–1.10 P = 0.108) was similar for the two study groups (Table 3).

**Table 3:** Primary outcomes in various follow up periods.

	Number of event (%)‡		Sitagliptin *vs*. Comparison	
Outcome	Sitagliptin	Comparison	HR (95% CI)†	*P*
**3 month follow up**				
Myocardial infarction	10 (1.8)	53 (1.9)	0.94 (0.48–1.84)	0.851
Ischemic stroke	5 (0.9)	15 (0.5)	1.67 (0.61–4.59)	0.322
Cardiovascular death	2 (0.4)	45 (1.6)	0.22 (0.05–0.91)	0.037
Primary endpoint§	16 (2.9)	108 (3.9)	0.74 (0.44–1.25)	0.262
**1 year follow up**				
Myocardial infarction	26 (4.8)	116 (4.2)	1.11 (0.72–1.69)	0.642
Ischemic stroke	12 (2.2)	44 (1.6)	1.35 (0.71–2.55)	0.360
Cardiovascular death	10 (1.8)	91 (3.3)	0.54 (0.28–1.04)	0.066
Primary endpoint§	45 (8.2)	233 (8.5)	0.96 (0.70–1.32)	0.802
**All course**				
Myocardial infarction	4.95	4.61	1.07 (0.72–1.59)	0.738
Ischemic stroke	2.60	1.98	1.30 (0.75–2.26)	0.346
Cardiovascular death	2.53	3.89	0.65 (0.39–1.10)	0.108
Primary endpoint§	9.50	9.70	0.97 (0.73–1.29)	0.849

^†^ adjusted for propensity score

^§^ anyone of myocardial infarction, ischemic stroke, or cardiovascular death

^‡^ Number of events per 100 person-years during the all-course follow-up

**Fig 3:**
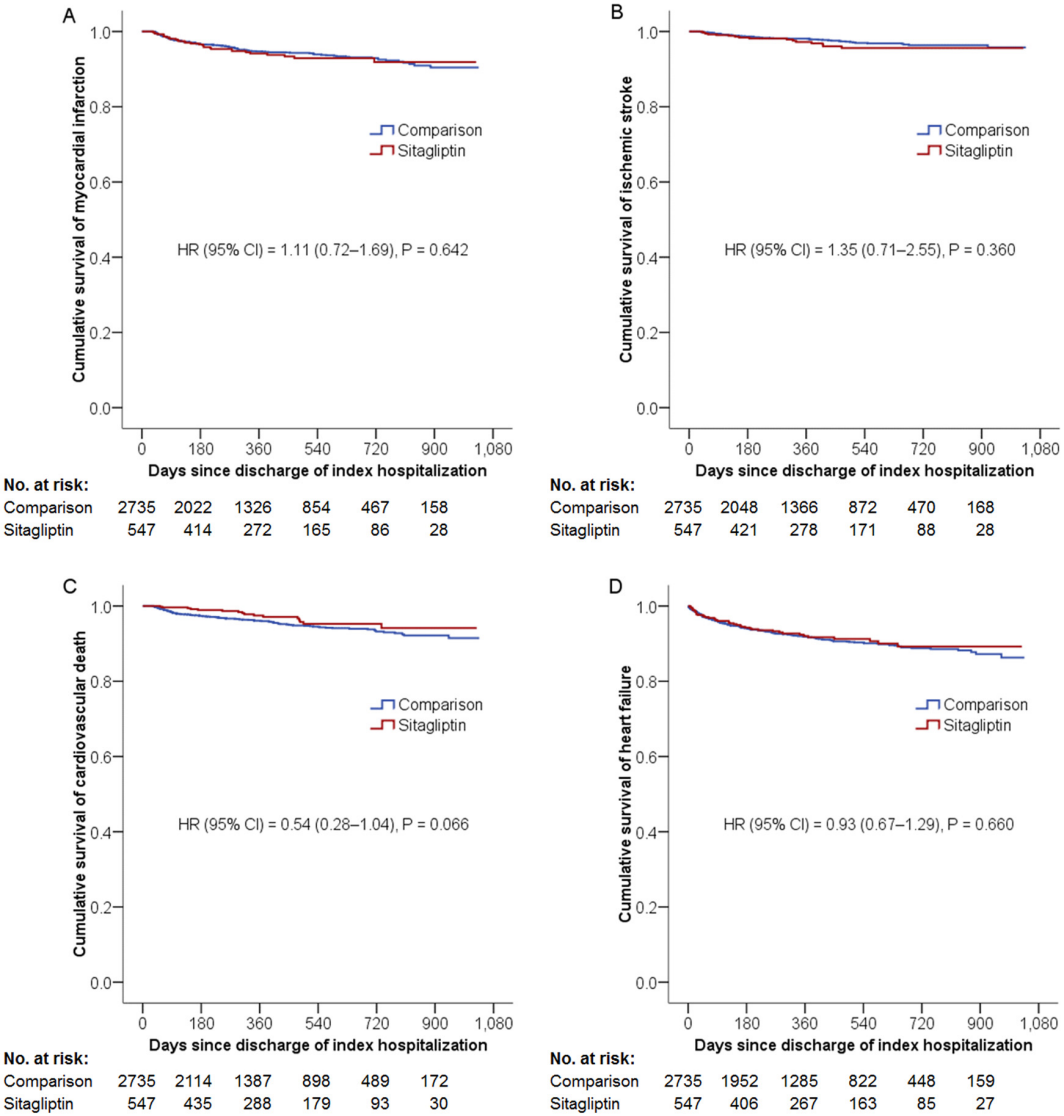
Cumulative Kaplan-Meier survival estimates of the time to individual components of the primary composite endpoint and heart failure hospitalization. The sitagliptin and comparison groups had similar incidence of individual components of the primary composite endpoint, such as AMI (Panel A), ischemic stroke (Panel B), cardiovascular death (Panel C), and hospitalization for heart failure (Panel D).

As for secondary outcomes, the sitagliptin group had similar risks of all-cause mortality, hospitalization for heart failure (HF), or percutaneous coronary revascularization with an HR of 0.82 (95% CI, 0.61–1.11, P = 0.195), 0.93 (95% CI, 0.67–1.29, P = 0.660), and 0.93 (95% CI, 0.75–1.14, P = 0.473) respectively, compared to the non-sitagliptin group (Fig 3). Subgroup analysis revealed that sitagliptin use was not associated with increased risk of heart failure hospitalization in patients with previous history of heart failure (HR = 1.05; 95% CI, 0.71–1.56, P = 0.809) or without it (HR = 0.73; 95% CI, 0.40–1.33, P = 0.304).

### Safety Outcomes

The sitagliptin and non-sitagliptin groups did not differ significantly with respect to incidence of hyperglycemia complications (0.95 and 0.54 per 100 person-years, HR = 1.78; 95% CI, 0.70–4.50, P = 0.227). The incidence of hypoglycemia was also similar across the two study groups (1.44 and 1.19 per 100 person-years; HR = 1.22; 95% CI, 0.59–2.52, P = 0.597). There were no significant differences in the incidence of pancreatitis between the two groups (Table 4).

**Table 4:** Secondary outcomes (all course).

	Number of event‡		Sitagliptin *vs*. Comparison	
Outcome	Sitagliptin	Comparison	HR (95% CI)†	*P*
**Other cardiovascular outcomes**				
Non-fatal myocardial infarction	4.62	4.11	1.12 (0.74–1.68)	0.598
Non-fatal ischemic stroke	2.44	1.94	1.24 (0.71–2.19)	0.453
Death from any cause	7.74	9.50	0.82 (0.61–1.11)	0.195
Heart failure	7.07	7.63	0.93 (0.67–1.29)	0.660
Percutaneous coronary revascularization	19.56	21.36	0.93 (0.75–1.14)	0.473
**Safety outcomes**				
Any pancreatitis	0.16	0.26	0.61 (0.08–4.87)	0.640
Acute pancreatitis	0.16	0.26	0.61 (0.08–4.87)	0.640
Chronic pancreatitis	0	0	NA	NA
Hypoglycemia	1.44	1.19	1.22 (0.59–2.52)	0.597
DKA, HHS	0.95	0.54	1.78 (0.70–4.50)	0.227

† adjusted for propensity score; NA = not applicable; DKA = diabetic ketoacidosis; HHS = Hyperosmolar hyperglycemic state; NA = not applicable due to no event was observed

‡ Per 100 person-years

## Discussion

In this population-based cohort study, we demonstrate that sitagliptin use was not associated with increased risk of primary composite cardiovascular outcomes among AMI patients, when compared to subjects who did not use sitagliptin. Analysis of individual components of the primary cardiovascular outcomes (AMI, ischemic stroke, and cardiovascular death) found no significant difference between these two groups at one-year and all-course follow up. Secondary outcome analysis demonstrated that the sitagliptin and comparison groups had a similar incidence of heart failure hospitalization, pancreatitis, hypoglycemia episodes and complications of hyperglycemia. The two groups also had similar likelihood of receiving a subsequent percutaneous coronary revascularization. To date, the cardiovascular effects of DPP-IV inhibitors have not been confirmed; our results suggest sitagliptin use is not associated with increased cardiovascular risks.

Our study aimed to evaluate the safety profile of sitagliptin in AMI patients on a population-based, nationwide scale. Compared with other studies [12,13], we examined only subjects who had a recent episode of myocardial infarction hospitalization, making our cohort at a much higher risk than patients in other studies. With a mean follow-up period of 14 months, 10% of our study subjects developed a primary endpoint, making our results invaluable for patients who are at high risk for adverse cardiovascular events. Researchers from the Examination of Cardiovascular Outcomes with Alogliptin versus Standard of Care (EXAMINE) trial reported a primary endpoint rate of more than 11%, but found no increased risk associated with alogliptin use when compared to placebo in patients with recent AMI or unstable angina [25]. Our results are in agreement with those of the EXAMINE trial, suggesting that DPP-IV inhibitors are safe for patients who have a high risk of adverse cardiovascular events. Notably, the sitagliptin group in our study had a reduced rate of cardiovascular death at three-month follow up, but not at one-year and all-course follow up (Table 3). Due to the nature of multiple testing and the possibility of type one error, the protective effect shown here should be interpreted with caution. Further research is warranted to elucidate this potentially beneficial effect.

Previous study from our group has indicated an association between sitagliptin use and increased risks of recurrent AMI and percutaneous coronary revascularization among type 2 diabetic patients with chronic kidney disease after AMI, especially in the end-stage renal disease subgroup [16]. The seemingly opposite conclusion reached by our previous paper is likely the result of difference in study population, in which all subjects had kidney function impairment and more than half diagnosed with end-stage renal disease. On the other hand, subjects with chronic kidney disease accounted for only 8% of the study cohort in the present study, and patients with end-stage renal disease were excluded. The drastic difference in renal function and other clinical characteristics may explain such contrasting results from the two studies.

Our results suggest that the use of sitagliptin was not associated with an increased incidence of heart failure hospitalization in the overall cohort, whether in patients with or without previous history of CHF. So far, a causal relationship between DPP-4 inhibitors and CHF has not been established. The recent SAVOR trial found an increased likelihood of hospitalization for heart failure in the Saxagliptin group [14,26]. Moreover, in a retrospective study using data from a U.S. commercial insurance claims database, Weir and colleagues showed that sitagliptin is associated with an increased risk of heart failure hospitalization in diabetic patients with incident heart failure [15]. Unlike the retrospective study conducted by Weir et. al., our research employed a prospective approach, enrolling patients who had a recent AMI, with well-matched baseline characteristics between groups, and a stricter definition of sitagliptin use that required patients to receive 90 consecutive days of treatment. With this rigorous study design, our results certainly provide a strong piece of evidence to the current research field.

In light of the controversy surrounding the safety of DPP-4 inhibitors, a well-designed, randomized double-blinded clinical trial is needed. The Trial Evaluating Cardiovascular Outcomes with Sitagliptin (TECOS) is an ongoing multinational clinical trial aiming to evaluate the efficacy of sitagliptin for reducing cardiovascular risk in patients with type 2 diabetes who have documented vascular disease in the coronary, cerebral, or peripheral arteries [27]. Although the TECOS trials enrolled patients who were at elevated cardiovascular risk, our study is unique in that only subjects who suffered a recent AMI were included. Therefore, our study provides an important source for evaluating sitagliptin safety in post AMI patients.

Our study has several strengths, including our prospective study design in which all patients who developed AMI were included and their outcomes analyzed. Moreover, after adjustment by propensity scoring, the sitagliptin and comparison groups are well matched in clinical characteristic. Lastly, we included only diabetic patients with recent AMI, who are at very high cardiovascular risk, making our study an invaluable source in this area of clinical research.

### Study Limitations

Our study has several limitations. First, the common confoundings of patient information were absent in our study, such as family history of cardiovascular disease, smoking, body mass index, or lipid profile. The two patient groups may differ in unmeasured ways. To avoid this bias, we utilized propensity score to balance every clinical characteristics between the two cohorts. We adapted the strictest enrollment criteria enrollment, which only included patients with acute myocardial infarction to ensure very-high cardiovascular risks existing between sitagliptin group and comparison group. We believe the methodologies used in this study are valid. Second, coding error may exist in a database. Our study based its patient enrollment and outcome measurement on NHIRD in-patient diagnosis codes. Since these diagnoses were related to insurance reimbursement, its accuracy is regularly audited by the NHI Bureau. False reimbursement claims would result in substantial penalties, making coding errors less likely to take place. The accuracy and validity of NHIRD data were also confirmed by previous study [18–20]. Third, due to the fact that sitagliptin was not available in Taiwan until March 1st, 2009, our study has a mean follow-up period of 14 months, and trials of longer duration may be needed to study long-term outcomes. This limitation also prevents us from tracking long-term patient history such as duration of diabetes. However, recurrent adverse cardiovascular events are more likely to take place within 1 year after AMI than in other time frames. The duration of this study should be sufficient to analyze association between sitagliptin use and cardiovascular outcomes [28]. Finally, our study is based on the assumption that patients are completely compliant with physician’s orders.

## Conclusion

In conclusion, this nationwide, population-based cohort study demonstrated that in type 2 diabetes patients with recent myocardial infarction, the use of sitagliptin is not associated with increased risk of composite adverse cardiovascular outcomes, including myocardial infarction, ischemic stroke, and cardiovascular death. Sitagliptin did not increase the risk of heart failure hospitalization, either. Our results can be used to help guide clinicians in formulating an optimal therapy for diabetic patients at very high risk of adverse cardiovascular events.

## Supporting Information

S1 Table ICD-9-CM code used for diagnosis in the current study. (DOCX)
