# Supplementary material for: Cardiovascular Outcomes of Sitagliptin in Type 2 Diabetic Patients with Acute Myocardial Infarction, a Population-Based Cohort Study in Taiwan
Source: PLoS One. 2015 Jun 26;10(6):e0131122. doi: 10.1371/journal.pone.0131122 (PMC4482692; doi:10.1371/journal.pone.0131122)
Supplement: S1 Table — (DOCX) [file pone.0131122.s001.docx]

S1 Table. ICD-9-CM code used for diagnosis in the current study

| Variable | Source | Code |
| --- | --- | --- |
| Myocardial infarction | ICD-9 CM | 410 |
| Chronic kidney disease | ICD-9 CM | 585 |
| Ischemic stroke | ICD-9 CM | 433–435 |
| Neuropathy | ICD-9 CM | 3572, 2496, 2506 |
| Retinopathy | ICD-9 CM | 2505 |
| Coronary artery disease | ICD-9 CM | 413,4140 |
| Chronic obstructive pulmonary disease | ICD-9 CM | 490–496 |
| Peripheral arterial disease | ICD-9 CM | 440.0, 440.2x, 440.3x, 440.4, 440.9, 443.9, 444.2, 444.22, 444.8, 444.81, 445.0, 445.02, 250.7x, 707.1x |
| Hypertension | ICD-9 CM | 401–405 |
| Heart failure | ICD-9 CM | 428 |
| Dyslipidemia | ICD-9 CM | 272 |
| Malignancy | ICD-9 CM | 140–208 |
| Acute pancreatitis | ICD-9 CM | 5770 |
| Chronic pancreatitis | ICD-9 CM | 5771 |
| Hypoglycemia | ICD-9 CM | 2510, 2511, 2512, 2508, 2498 |
| DKA or HHS | ICD-9 CM | 2501, 2502, 2503 |

DKA = diabetic ketoacidosis; HHS = Hyperosmolar hyperglycemic state.
